# Developing a Multi-variate Logistic Regression Model to Analyze Accident Scenarios: Case of Electrical Contractors

**DOI:** 10.3390/ijerph17134852

**Published:** 2020-07-06

**Authors:** Pouya Gholizadeh, Behzad Esmaeili

**Affiliations:** Sid and Reva Dewberry Department of Civil, Environmental and Infrastructure Engineering, Volgenau School of Engineering, George Mason University, 4400 University Drive, MS 6C1, Fairfax, VA 22030, USA; besmaeil@gmu.edu

**Keywords:** occupational accident analysis, electrical contractors, predicting fatality rates, multi-variate logistic regression

## Abstract

The ability to identify factors that influence serious injuries and fatalities would help construction firms triage hazardous situations and direct their resources towards more effective interventions. Therefore, this study used odds ratio analysis and logistic regression modeling on historical accident data to investigate the contributing factors impacting occupational accidents among small electrical contracting enterprises. After conducting a thorough content analysis to ensure the reliability of reports, the authors adopted a purposeful variable selection approach to determine the most significant factors that can explain the fatality rates in different scenarios. Thereafter, this study performed an odds ratio analysis among significant factors to determine which factors increase the likelihood of fatality. For example, it was found that having a fatal accident is 4.4 times more likely when the source is a “vehicle” than when it is a “tool, instrument, or equipment”. After validating the consistency of the model, 105 accident scenarios were developed and assessed using the model. The findings revealed which severe accident scenarios happen commonly to people in this trade, with nine scenarios having fatality rates of 50% or more. The highest fatality rates occurred in “fencing, installing lights, signs, etc.” tasks in “alteration and rehabilitation” projects where the source of injury was “parts and materials”. The proposed analysis/modeling approach can be applied among all specialty contracting companies to identify and prioritize more hazardous situations within specific trades. The proposed model-development process also contributes to the body of knowledge around accident analysis by providing a framework for analyzing accident reports through a multivariate logistic regression model.

## 1. Introduction

In 1931, safety theorist Herbert William Heinrich described safety problems as relating to three elements: environment (i.e., safe or unsafe states); decision space (i.e., safe or risky human acts); and the probability that an accident happens given the risky action of humans in an unsafe state [1]. While Heinrich’s concepts have shaped much of the industrial and occupational safety considerations of the last ninety years [2,3], current fatality and injury trends within various industries demonstrate that these contributing elements have not been sufficiently controlled or considered to keep workers safe. More than 5000 workers have died while working and about 2.8 million workers were injured on jobsites in 2017. Almost one in every five fatalities has occurred in the construction industry [4]. These statistics further emphasize that new solutions to managing construction risk appear necessary.

Many studies have leveraged Heinrich’s parameters to model occupational accidents and improve worksite safety for workers [5,6,7,8,9,10]. For instance, Barkan et al. [11] integrated signal detection theory into Heinrich’s framework to design and execute experiments to study the effects of several factors (e.g., giving positive feedback for good behaviors while imposing punishment for unsafe behaviors) on the probability of choosing a risky task in an unsafe state (i.e., miss rates). Furthermore, to tailor Heinrich’s ideas to construction accident investigations, Abdelhamid and Everett [12] proposed an accident root causes tracing model (ARCTM) which emphasizes unsafe environment/acts as the main root cause of accidents; ARCTM’s application is intended to identify accident causes to prevent accident recurrences. At their root, these applications of Heinrich’s theory demonstrate the desire to understand the circumstances under which workers—especially construction workers—are more prone to fatalities in occupational accidents, in order to improve safety performance.

However, defining the circumstances of events (i.e., unsafe conditions/acts) can be challenging. Abdelhamid and Everett [12] suggested that any physical layout, status of materials, tools, and so on, that are in violation of safety standards are a type of unsafe condition. Another study defines an unsafe act as “a violation of an accepted safe procedure which could permit the occurrence of an accident” [13] (p. 5). In the dynamic and diverse domain of construction, such specifications are too general to be useful to safety practitioners. Furthermore, these generically defined factors do not exist in a vacuum, so the interactions between environment, behavior, and probability make identifying and proactively managing risk a compounding puzzle for practitioners.

To address this issue, this study builds a multivariate statistical model to identify which elements combine to contribute to more serious accident scenarios (i.e., combinations of project’s characteristics, worker’s tasks, and accident factors) among those performing a specific trade in the construction industry. To select a trade for analysis, the authors evaluated the specialty trade contractors’ classifications within the North American Industry Classification System (NAICS) and found that electrical contractors faced the largest increase in the number of fatalities in recent years [14]. In addition, electrical contractors face a wide range of accident types (e.g., such as electrocution, fall, struck-by, and caught in/between) that represent multiple accident scenarios. The variety of accident scenarios enabled the research team to better measure the impact of each individual accident factor. Furthermore, considering the large number of registered firms as electrical contractors and the large number of workers employed by these specialty contractors [4], the research team decided to consider electrical contractors as a prime candidate for the analysis in this study.

In this study, we used historical data about past accidents among electrical contractors to provide a quantitative representation of the unsafe conditions/acts that existed on the construction sites, with the severity of injuries serving as the metric to evaluate the safety performance. Then, using a thorough logistic regression modeling framework, the research team iteratively analyzed the impact of individual accident factors—as described in the accident reports—on the accidents’ outcomes; here, the severity of injury among the electrical contractors. As a logistic regression model determines which factors in a pattern have a significant effect on the dependent variable, this approach allowed our team to determine which accident factors contribute significantly to the injury pattern. Given that an accident pattern captures factors describing both unsafe environments and unsafe acts on a construction site, by identifying significant contributory factors affecting accident severity, our model detects high-priority factors that may be preemptively mitigated to prevent or ameliorate accidents. Such knowledge of accident patterns helps safety managers prioritize addressing these conditions in the safety planning and resource-assignment phase of future projects, and therefore supports worker safety while saving safety practitioners time and resources.

The rest of the paper is organized as follows: (1) the background section discusses findings of a literature review regarding the causes and circumstances of occupational accidents among electrical contractors; (2) the methodology section details the logistic regression modeling process, where data analysis and modeling were done using the R language [15]; (3) the results of each modeling process step are demonstrated for the case of electrical contractors, and the hazardous accident patterns observed in this trade are outlined; and lastly, (4) the important findings of the paper are discussed and summarized.

## 2. Background

### 2.1. Analyzing Accident Severity against Contributing Factors

Investigating incident severity through statistical modeling and data-mining techniques has been a popular approach among accident-prevention studies [16,17,18,19]. A data mining method, association rules, have been used by Liao and Perng [20], and Cheng et al. [21] to analyze construction accidents to discover potential associations among accident factors. Liao and Perng [20] found that in civil engineering projects worker’s age (i.e., between 45–54) and time of service (more than 365 days) are most associated with higher probabilities of fatal injuries. In building construction projects, however, the study found that the worker’s salary (i.e., more skilled workers) and day of the week (Mondays and Tuesdays) are more associated with higher fatalities. Cheng et al. [21] applied association rules to investigate the main factors that are associated with falls in civil engineering and building construction projects. They revealed that “failure to install a work platform or protection when working in a high place, workers’ horizontal movements, and failure to use personal protection equipment when at work” are the top three factors contributing to falls in both types of projects (p. 443). 

Another popular statistical modeling method to explain relationships between accident variables and the outcome of the incident is logistic regression [22,23,24]. Many accident analyses studies have utilized variations of logistic regression models. For instance, Al-Ghamdi [25] applied logistic regression on police reports to examine the effects of several variables on the severity of injuries and revealed that location and cause of accident are the most significant factors. In another study, Yan et al. [26] analyzed the probability of rear-end accidents using Florida traffic accident data to identify the significance of risk factors such as road environment features (e.g., number of lanes, road surface condition) and driver/vehicle traits (e.g., drivers age, vehicle type) on this type of accident. In a study more relevant to construction safety, Harb et al. [27] investigated three years of work-zone crash data from Florida using multiple logistic regression models; the authors found roadway geometry, age and gender of the driver, usage of drugs and alcohol, lighting and weather conditions can all be major risk factors of work-zone crashes. Chau et al. [28,29] reviewed 880 cases in the French construction industry to study the effects of the individual characteristics of workers on accident occurrence. The results indicated that sleep disorders and young age significantly contribute to higher injury rates.

Savolainen et al. [30] performed a comprehensive review of studies related to the application of logistic models in accident analysis; their work shows that among various modeling frameworks, discrete outcome models such as ordered probit and unordered (i.e., nominal) logit models have been applied the most. The choice between these models mainly depends on the nature of the response variable (i.e., degree on injury). When the accidents are classified with either fatal or nonfatal outcomes, binary outcome models become a viable choice for analysis. For instance, in an attempt to quantify the effects of four different street patterns on road accident injury risks—controlling for other parameters such as driver’s age, condition, and so on—Riffat and Tay [31] analyzed 22,704 crashes using a binary outcome logistic regression model; the study showed that some patterns such as lollipops and loops can be marginally safer when compared to the traditional gridiron pattern. Another study by Peek-Asa et al. [32] investigated the impact of teenager drivers’ age on the degree of injury on rural and urban roads. This study found that among teenage drivers the odds of a fatal/severe injury were almost five times higher in non-urban areas than urban environments. The authors concluded that the higher fatality rates in rural areas could be associated with road conditions, uncontrolled intersections, narrower two-lane roads and less visibility. Each of these types of accident-severity analyses attempts to detect accident factors with significant impacts on accident severity, and they can all lead to a better understanding of the latent causes of accidents, as well as to the design of better safety solutions.

While the occupational safety literature is rich in the field of construction, severity-analysis studies based on empirical data are very limited compared to transportation accident studies. The lower number of accidents and fewer data sources, as well as the unique conditions of construction sites—as compared to the relatively ubiquitous conditions in traffic crashes—reasonably explains this gap in the literature. Furthermore, the majority of safety studies/programs in construction have focused on developing methods to reduce the frequency of accidents and not the severity of their outcomes [33]. This study addresses these knowledge gaps by analyzing the severity of construction accidents through a multi-variate model. Another limitation, even among crash-injury analyses, is that many details of the modeling process are usually missing from these studies. Thus, discussing the results of these models without describing the details can hurt the final conclusions, as logistic models can be very sensitive to their assumptions. Therefore, the authors provided a detailed description of modeling steps that can be replicated in future safety studies.

### 2.2. Occupational Incidents among Electrical Contractors

Construction is one of the largest industries in the United States. According to the Bureau of Labor Statistics, more than nine million people worked in this industry in 2018 [34]. The construction industry also consists of a wide range of professions and specialties, and therefore a large number of unique environments around each project. To limit the scope of analysis, the authors decided to focus on only one specialty trade, electrical contractors.

According to the Statistics of U.S. Businesses (SUBS), in 2016 more than 144,000 establishments and firms were registered as electrical contractors, making this subindustry the second largest in business count among all specialty trades [35]. In 2017, this trade also had the third highest number of fatalities among all 18 specialty trades within the industry [34]. To better understand accident patterns among electrical contractors, the authors have conducted an in-depth literature review related to occupational incidents in this trade.

Studies investigating the nature of accidents among electrical contractors are minimal [36]. In one early study, an examination of the mortality patterns of 31,068 U.S. members of the International Brotherhood of Electrical Workers (IBEW) working in the construction industry between 1982 to 1987 showed an elevated proportion of mortality due to causes such as leukemia, brain tumors, melanoma skin cancer, and diseases caused by asbestos (e.g., lung cancer) [37]. However, their study reviewed mostly long-term causes of injuries—such as cancer and heart disease—and therefore did not cover day-to-day hazards and occupational injuries on construction sites.

Rossignol and Pineault [38] investigated fifty-seven electrocution accident reports that resulted in a fatality in Quebec during the period of 1981–1988. The authors defined three phases as pre-electrocution, electrocution, and post-electrocution, and they then set out seven descriptors for the first two phases. The authors used factor analysis to classify the seven pre-defined descriptors. Results of the factor analysis showed more than 90% of cases were covered with only two factors: (1) twenty-six electrocutions occurred during an indoor electrical task performed in direct contact with a source of less than 10,000 volts; and (2) twenty cases happened while performing non-electrical outdoor tasks in indirect contact with a source of more than 10,000 volts. This clear distinction raised questions about the efficiency of safety trainings where around 43% of accidents occurred in non-electrical tasks. The authors suggested a shift in safety strategies from training to elimination or modification of hazard sources. While the outcomes were interesting, the study suffered limitations from a relatively small and old database of cases from outside the United States.

In a study that considered age, company size, experience, tasks performed, source of injury, and accident causes as dependent variables, Chi et al. [39] performed a chi-square automatic interaction detector (CHAID) analysis of 250 fatal electrocution accidents to classify accidents into seven hazard-pattern scenarios. Among all these predictors, only source of injury and accident causes were indicated to be significant in classifying accident scenarios. Results of the study show that if the source of injury was energized equipment, then almost all electrocutions occurred due to direct contact; for other electrical sources, direct or indirect contact would happen relatively equally. As the authors indicated, the main limitations of this study were inconsistency in reporting accidents and using only two predictors in the analysis, along with the relatively small number of observations.

Considering the high proportion of fatalities due to electrocution in construction even after safety controls have been employed for decades, Zhao et al. [40] examined 486 recommended controls from 132 Fatality Assessment and Control Evaluation (FACE) accident reports to: (1) evaluate the effectiveness of electrical safety recommendations given by NIOSH experts; and (2) assess safety knowledge in the construction industry. Accident reports were coded using the hierarchy of controls: elimination, substitution, engineering, administration, and personal protective equipment. The authors also defined a variable to represent safety knowledge based on the number of recommendations for each accident. They compared these two variables by three important parameters for electrical safety—construction type (residential, commercial, etc.); occupation (electrician, and non-electrician); and electrical condition (low voltage and high voltage)—and found that the number of suggested safety practices was slightly higher for non-electrical workers, and control measures for high-voltage hazards, were statistically less effective than those for low-voltage hazards. The effectiveness of controls was not statistically different by construction type or occupation. Another interesting finding was that behavioral controls in electrical hazard mitigation are overemphasized and more attention should be paid to effective control measures, such as elimination. While the paper made several valuable contributions, it suffered the limitation that FACE reports were collected in multiple decades and were not randomly selected. Therefore, the assumption that the recommendations provided in those reports are common practices in the construction industry would not be correct.

To better understand the chain of decision mistakes that lead to an electrical accident, Zhao et al. [41] examined 144 FACE accident reports. Considering 12 common decision mistakes (e.g., failure to lockout/tagout) as independent variables and 19 pre-defined factors (e.g., project type, safety training, etc.) as predictors, the authors classified accident reports using Exhaustive CHAID, a type of classification tree. The analysis detected five features of work (i.e., group of activities and sequences) and decisions that could prevent electrocution accidents. One main finding of the study was that the sooner a decision is made, the better it can mitigate the risk of electrical accidents. The results of the study can help safety managers find critical decision points in different situations in order to suggest effective safety controls before the construction phase. Using the same database, Zhao et al. [42] studied electrical injuries by considering the interactions among humans, technology, social structure, and environment as a sociotechnical system. By conducting latent class analysis and multiple correspondence analysis, the authors identified three sociotechnical systems in the construction industry: residential-building; heavy and civil-engineering; and non-residential-building construction. The authors provided specific recommendations for each system to reduce the risk of electrocution injuries. However, both of these studies were conducted on a small number of fatal accident reports; for this reason, a severity analysis could not be done on such data.

In another study, Gholizadeh and Esmaeili [43] studied the effects of different accident types and project end uses on the cost of injury among electrical contractors using robust hypothesis testing methods (i.e., Welch-type procedure, extension of Yuen’s method, and percentile bootstrapping). The results of the study confirmed that robust hypothesis testing approaches can be successfully implemented on safety data even when the assumptions of conventional test statistics are violated. The findings showed that various event types and project end-uses can impact the cost of injuries among electrical contractors: caught in/between and exposure to electricity accidents can, on average, lead to higher injury costs than fall to lower levels and struck-by objects/equipment accidents. For a project’s end-use, the outcomes indicated that injuries that occurred in nonbuilding projects are significantly costlier than those that occurred in building projects.

The results of the literature review indicated several limitations in previous studies on the occupational health and safety of electrical contractors: (1) the majority of studies have focused only on fatalities and ignored other incident outcomes; (2) most of the studies investigated electrical incidents and not all types of possible hazards (e.g., fall, struck by) among electrical contractors; and (3) there is an absence of robust statistical analysis to support inferences made from incident databases—severity-analysis studies (i.e., using statistical models to explain and predict the outcome of an accident) are limited in the construction safety domain. On the other hand, researchers have been able to enhance our understanding of traffic accidents and their consequences by applying logistic regression models on traffic crash accident data. The authors believe that construction safety managers can also benefit from such analysis by identifying more severe accident scenarios. While the modeling method used in this study has been successfully applied to traffic accident data, its application to construction accident data was not guaranteed before this study. Our findings show that empirical data available publicly through organizations such as the Occupational Safety and Health Administration (OSHA), while they may need improvements, are suited for such analysis, and that statistical models can be built upon construction accident data to find more severe patterns and prioritize managing them. Therefore, this study aims to address these limitations by using a multivariate logistic model to analyze accidents in the OSHA’s Integrated Management Information System (IMIS) accident database affecting electrical contractors.

## 3. Materials and Methods

To attain the research objectives, the research team first acquired reliable national data on occupational incidents involving electrical contractors from OSHA’s online database of catastrophic accidents. The authors then conducted a thorough content analysis to ensure consistency among variables, to reduce any ambiguity in reported values, and to prepare data for statistical analysis. As the most severe accidents end in a fatality, this study uses fatality rates to describe accident severity. Thus, to investigate and explain the relationship between factors contributing to accidents and the degree of accident injuries, this study executed a multivariate logistic regression model that estimates the fatality rates of different accident scenarios occurring among electrical contractors. The ability to consider several factors in one model and interpret the final coefficients in terms of adjusted odds ratios (i.e., controlled for other factors) makes multivariate logistic modeling a suitable approach for severity analysis. The rest of this section is devoted to explaining each of these steps.

### 3.1. Incident Database and Programming Language

The authors collected 621 accident reports involving electrical contractors between 2007 to 2013 from OSHA’s IMIS online database. In total, 689 employees were injured in these incidents while performing their jobs on construction projects. The OSHA IMIS database has been successfully used by previous researchers to study occupational incidents [44,45,46]. Within this database, a summary of each accident, as reported by OSHA inspectors, appears along with variables that describe the accident (e.g., event type, source and cause of injury), its context (e.g., project end-use, type, and cost), and its consequences (e.g., nature and degree of injuries, and injured part of body). As this study’s concern is contributing factors, the analysis only considers variables that manifest before an accident occurs; therefore, variables such as nature of injury (e.g., fracture, burn), part of body (e.g., head, trunk), and event type (e.g., fall, struck by, exposure to electricity) are excluded from the modeling process in this study because these elements are all characteristics of the accident after the accident occurred and therefore do not represent contributory factors to accident severity.

The data analysis and model development steps were done in R environments [15]. R is an open source language for statistical computing and graphics which provides a broad range of statistical techniques such as classical statistical tests, classification, clustering, time series analysis, and linear and non-linear modeling. R environments can run on Windows, MacOS and Unix platforms.

### 3.2. Odds Ratio

To better understand the associations between each significant covariate and the target variable (i.e., degree of injury) and to interpret the likelihood of a fatal accident in different situations, the unadjusted odds ratios of fatal accidents were calculated at this point. Odds ratios can compare the magnitude of different risk factors over an outcome [47]. For example, consider a comparison of the odds of a fatal injury among all accidents where the source of injury is either a vehicle or a machinery. The odds ratio for the effect of the vehicle category in this example can show the magnitude of its effect on the fatal injuries; an odds ratio of 4 means that the chance (i.e., odds) of a fatal injury is four times more where the source is a vehicle compared to cases where the source is a machine. These ratios can be compared to the adjusted ones once the final model is selected, as such a comparison reveals the effects of controlling factors on odds ratios. Significant changes among unadjusted and adjusted ratios would suggest high correlations between accident factors, and further encourages the use of logistic models to achieve adjusted ratios.

### 3.3. Logistic Regression

While using chi-square tests can show the relationship between a single factor and the degree of injuries, these tests are unable to determine the effects of these variables in the presence of other factors. Logistic regression is a proper method to test the association between potential accident risk factors and a dependent variable [27]. As mentioned in the background section, many studies have used various types of logistic regression models to investigate potential associations in the field of accident analysis. While more advanced machine learning (ML) methods have emerged and been applied to accident studies in the past decade, logistic regression has some advantages over ML methods to justify its application in this study. First, the results of a regression model are easier to interpret. The model can be represented in one formula using only the independent variables and their coefficients. The coefficients of the model can directly determine important variables, along with the magnitude and direction of association between each independent variable (i.e., risk factor) and the dependent variable. This property is very important in studies where finding the relationship between risk factors and the dependent variable is as significant as the accuracy of the model’s predictions. This could be the main reason for the popularity of regression modeling in traffic accident analysis studies. Therefore, while some machine learning methods such as decision trees—and their variants such as random forests and gradient boosting trees—and support vector machines might provide better prediction accuracy, their lack of interpretability can be a disadvantage. Second, once the risk factors are identified, developing a logistic regression model is straightforward and, unlike machine learning methods, does not require tuning various hyperparameters. This quality makes logistic regression the first choice in many predictive studies and a valid baseline for other more complicated classifiers. Third, while methods such as association rules can be useful to find latent patterns in large data sets, these methods are inherently different from classification methods such as logistic regression modeling. As Freitas [48] has outlined, classification methods are about using the past data to predict the future; prediction is a non-deterministic task, and that’s why two different classification methods (e.g., logistic regression and decision trees) could generate different predictions on the same set of values. On the other hand, association rules are deterministic: every algorithm would produce the same set of rules, while some might be faster. One of the objectives behind modeling construction accidents in this paper is to use a reliable and well-defined model to predict fatality rates in common accident scenarios; association rules cannot be used for such predictions.

A logistic regression model can isolate effects and indicate which variables can explain the variability among accidents more accurately. Logistic regression models have been adopted widely in areas ranging from medicine [49,50] to the social sciences [51,52]. This section details several steps in developing and evaluating a multivariate logistic regression model.

Traditionally, building statistical models starts with selecting variables that can result in a parsimonious model (i.e., having as few variables as possible), explain the data, are stable, and can be generalized to unseen situations [53]. To develop such models, this study adopted the purposeful selection of variables procedure proposed by Hosmer et al. [54]. One main advantage of this approach is that it considers both the significance and change-in-estimate criteria when selecting final variables [55]. Each step will be explained here. One should note that to maintain the language of statistical modeling, accident factors are called “covariates” in this section.

**Figure 1:**
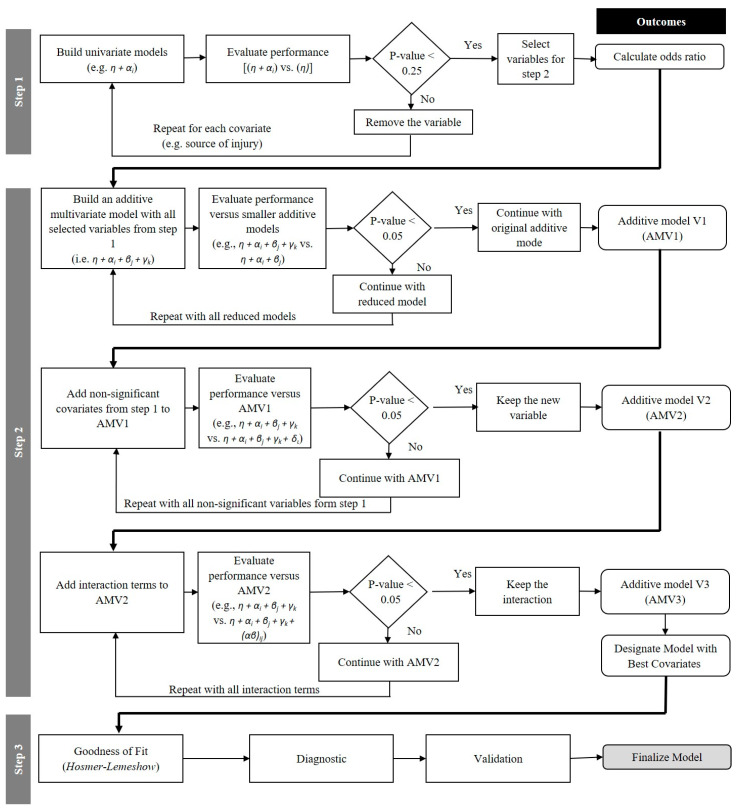
Three steps of building and validating a multivariate logistic regression model.

Three steps were taken to conduct this regression analysis (Figure 1):

- Step 1: Select variables and calculate odds ratios
- Step 2: Develop and adjust model
- Step 3: Assess and validate model

Step 1: Select variables and calculate odds ratios

A purposeful model-building process starts with building univariate (i.e., containing only one covariate such as project end-use or source of injury) models for each covariate and assessing their performance. The performance of each univariate model is calculated as the difference between its deviance and the deviance of a model with only the constant parameter (i.e., no covariate). The glm function from the stats package in R [15] was used to determine the deviances of models. The significance of difference (i.e., presented by G) is determined through the p-value of a chi-square test (i.e., the pchisq() function in R) with a degree of freedom equal to (d-1), where ‘d’ is the number of categories of the covariate. Within this framework, a significant result recommends the inclusion of the variable in the final model. As this is the first step, Hosmer et al. [54] recommended less conservative significance levels (i.e., 0.25 instead of 0.05) to include more variables in the model. In other words, this step allows less significant factors to remain in the model in order to analyze the effects of these apparently less significant factors on more significant ones in future steps. Factors that are neither significant at the 0.05 level nor have an effect on other factors would be excluded from the model eventually.

Table 1 presents the log likelihood ratio statistics (i.e., G) for five univariate models. Low values of G indicate that the difference between fatality rates among different categories of a factor are negligible and therefore the variable would not be very predictive in the model. The significance of G also depends on the degree of freedom (i.e., d.f.) which ultimately determines the *p*-values (i.e., the evidence against a null hypothesis: the smaller the p-value, the stronger the evidence to reject a null hypothesis). One can only check *p*-values and conclude whether the effect of a variable on fatality rates is significant or not. As the significant level in this step is 0.25, any value less than that is considered as significant at this step. Based on p-values of the chi-square tests, “end use” and “project cost” do not have significant effects on the probability of a fatal accident (even at the significance level of 0.25); in other words, the univariate models with these variables do not differ significantly from a model that has no covariates. The results indicate that the other three covariates are significant and should be considered in the multivariate model.

Step 2: Develop and adjust model

After finding the more important covariates (i.e., “source of injury,” “cause of injury,” “project type”), one can build an additive (i.e., no interaction) model and test the importance of individual covariates in this multivariate context using traditional significance levels (i.e., 0.05 in this study). The non-significant covariates are temporarily removed from the model—in the case of categorical covariates, all levels should be removed even if only one of the categories is not significant. Next, the deviance of the new/reduced model is compared to the deviance of the original multivariate model (i.e., likelihood ratio test). A large difference means that the removed variables—though independently not significant—have a considerable effect on adjusting the significant variables, and hence should be added back to the model. This process can be repeated several times to make sure that the necessary variables are included in the model. The last step in building a multivariate model includes the interaction effects. As with the single covariates, the interaction terms are added to the model one-by-one, and their effects are measured through the amount of deviance they can reduce. Thus, using this approach, significant interactions also remain in the final model. The modeling step is accomplished using, mainly, the generalized linear modeling [i.e., glm()] function with ‘binomial’ link in R [15].

As there are only eighteen possible models of interest based on the different combinations of these variables and their interactions, all of them are shown in Table 2. Three subscripts were used to reflect the structure of the data: let π_ijk_ be the probability of a fatal accident in the (i, j, k)-th group, where i = 1, 2, 3, 4, 5, 6 indexes “source of injury,” j = 1, 2, 3, 4, 5 presents levels of “project type” and k = 1, 2, 3, 4, 5, 6 indicates the categories under “cause of injuries”. These variables can produce 180 accident patterns (i.e., 6 × 6 × 5). However, 72 of these patterns have zero cases in the data and therefore should be excluded from analysis, leaving 108 covariate patterns for modeling. As all predictors are categorical, the authors decided to focus on fatality rates among these patterns instead of looking at each accident individually. Table 2 shows models in abbreviated notation, formulas for the linear predictor, the deviance, and the degrees of freedom. A unique ID number has also been assigned to each model for future references.

Using the deviance and degree-of-freedom from Table 2, one should start with the additive model with three covariates (i.e., model 11). Next, one covariate would be excluded from the model to check its effect on the other two covariates. The results of these tests are presented in Table 3 (i.e., a1, a2, and a3). The results reveal that the additive model with three variables (i.e., SoI + PT + CoI) represents a significant improvement over all the additive models with two factors (i.e., model 5, 6, and 7). In other words, while some levels of covariates are not significant (results are not shown), they have a significant effect on adjusting the other variables and therefore should remain in the model.

The next step is to investigate the interaction effects, which includes models with one, two, or three interaction terms. Three models (i.e., 12, 13, 14) in Table 2 have one interaction term. For example, model 12 includes the main effects of source of injury (SoI), project time (PT), and cause of injury (CoI), and the interaction between SoI and PT. The log likelihood tests in Table 3 (i.e., b1, b2, and b3) show that none of these models with one interaction is better than the three-factor additive model (i.e., model 11). One can also consider models involving two interactions between two factors, of which there are three (i.e., models 15, 16, 17). The results show that only one model (i.e., model 16) is marginally (*p*-value: 0.071) better than the additive model. Model 18, which includes all the interactions between each two variables, could not improve the additive model, and hence cannot be selected as a good model. Considering the p-values in Table 3, one can conclude that model 11 (SoI + PT + CoI) is the best model.

The last task in model development is to consider non-significant variables from step 1 in the context of the new multivariate model and check whether they can improve the performance of this model. Table 4 shows the results of such comparisons and declares that adding the non-significant variables, one-by-one and together, cannot lead to better results. For instance, adding end use to the model would reduce the model by four degrees of freedom while only reducing the deviance by 0.73, which is not even close to a significant improvement (*p*-value: 0.949). Therefore, the research team concludes that the additive model with three variables is the best multivariate model among the possible options.

Step 3: Assess and validate mode

After building a model by selecting covariates and tuning the model in a purposeful manner, one should investigate the probabilities that are produced by the model against true values in data. To do so, the last step in every statistical modeling process includes model assessment. This section will detail this study’s three main attempts to fulfill the requirements needed to assess our final model.

I. Goodness of fit

After building models and comparing deviances to determine covariates and build a parsimonious model, one needs to examine how well the data fits the final model. Lack of fit means that estimated coefficients are biased, odds ratios could be misleading, and future predictions are not accurate. To check fit, one can compare predicted values derived from the model to observed values to confirm that the fitted model is correct [56]. The Hosmer-Lemeshow (HL) statistic is a popular test for goodness-of-fit and has been used in several clinical studies [49,50,57]. The idea is to partition observations based on their estimated probabilities into g (usually 10 to represent deciles of risk) groups featuring approximately the same quantity of observations; here, the first group would represent the lowest probabilities of fatality, the next group would have larger estimated probabilities and so on, until the last group which includes the highest probabilities [58,59]. The HL statistic is then calculated by comparing the sum of probabilities to the number of observed values in each group. A chi-square test on this value with g-2 degrees of freedom can determine whether there is enough evidence to reject the hypothesis that data fits the model. The ‘hoslem.test()’ function from ‘ResourceSelection’ package in R [15] provides the test statistic and p-value of the HL test.

**Figure 2:**
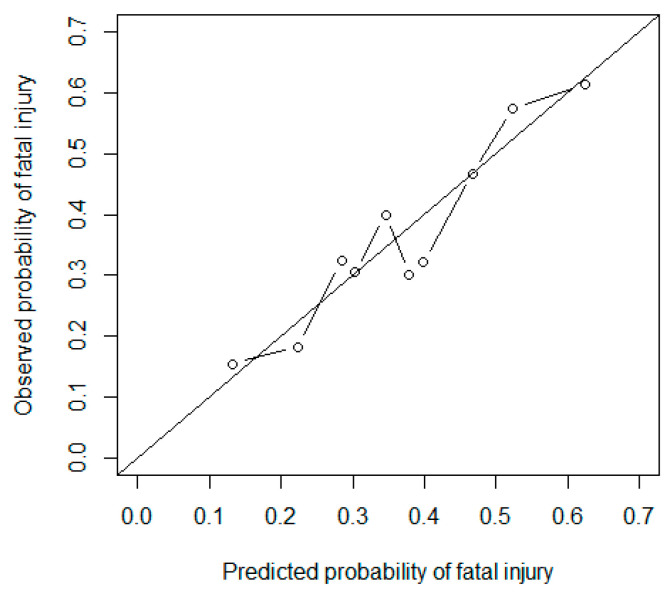
Observed and predicted probabilities of fatal injury in ten deciles of risk.

Table 5 shows the results of the Hosmer-Lemeshow tests when dividing the accident patterns into 10 groups. The value of Hosmer-Lemeshow’s goodness of fit computed for the frequencies in Table 5 is 5.85 when granted 8 degrees of freedom, with the corresponding *p*-value of 0.664. The large p-value indicates that the null hypothesis that the model fits the data cannot be rejected, demonstrating that the model fits the data quite well. A comparison of observed and expected frequencies in the 20 cells in Table 5 also shows close agreements in every decile of risk. For instance, within the highest risk decile (i.e., decile 10), the difference between both observed and expected values are within one point. Figure 2 compares the average of observed and predicted values in each decile, which again shows a close agreement in most deciles.

II. Diagnostics

Even a strong fit can be very sensitive to outlying and extreme-leverage points in the data [60]. So, while summary statistics such as the HL test can indicate the overall fit of a model to data with a single number, one still needs to check if the model fits over all covariate patterns. Pregibon [61] introduced a range of diagnostic measures for logistic models with binary outcomes. Two types of measures, based on the leveraged value of covariate patterns, are adopted in this study: (1) those that determine fit in each pattern (i.e., change in value of the Pearson chi-square, Δχ^2^, and change in the deviance, ΔD); and (2) those that can determine the amount of influence a pattern can have over other patterns (i.e., change in value of the estimated coefficients, Δβ).

For each covariate pattern, values of Δχ^2^ (and ΔD) are calculated as the difference between the Pearson chi-square (and deviance) values of the original model and the model when excluding observations in that pattern. As mentioned by Peng et al. [62], at the significance level of 0.05, and based on the critical value of the chi-square distribution with one degree of freedom (i.e., 3.84), changes more than four are considered large and demonstrate that the pattern in question contributes significantly to the disagreement between the observed and predicted values. Large values of Δβ also indicate that estimates are not stable. Large values of Δχ^2^ or ΔD accompanied by large changes in coefficients can signal that a covariate pattern is an outlier and should be investigated in more detail.

**Figure 3:**
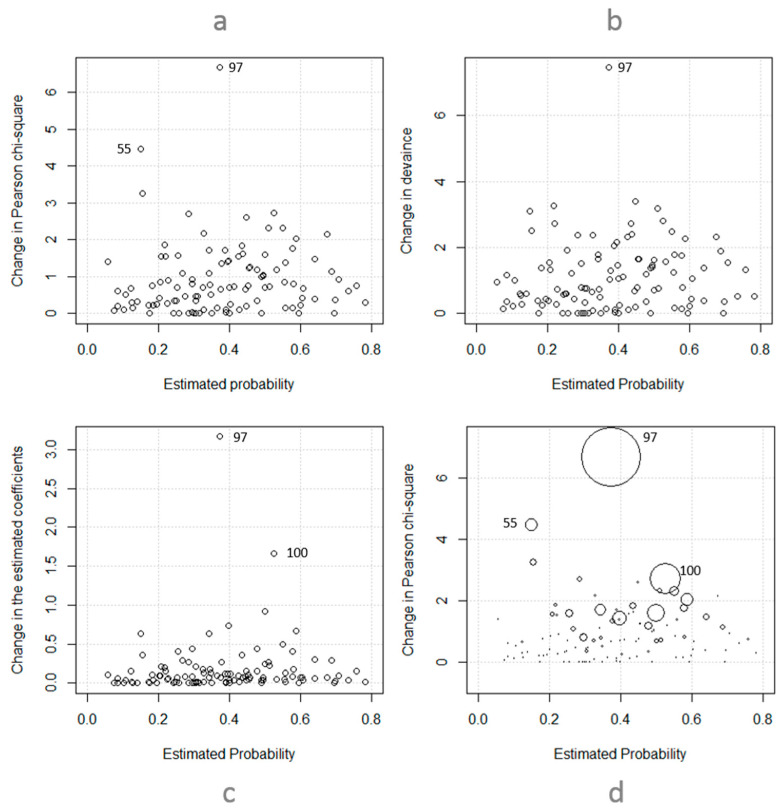
Diagnostic measures for the logistic model, (**a**) Change in Pearson Chi-square when removing individual covariate patterns from data, (**b**) Change in deviance when removing individual covariate patterns from data, (**c**) Change in the estimated coefficients when removing individual covariate patterns from data, (**d**) Combination of a and c: change in Pearson Ci-square (y-axis) and estimated coefficients (size of circles) when removing individual covariate patterns from data.

Figure 3 and Table 6 show diagnostic measures for covariates in the final model. Regarding the poorest fit, covariate pattern 97 (i.e., SoI: “parts and material,” PT: “alteration or rehabilitation,” CoI: “other”) and 55 (i.e., SoI: “machinery,” PT: “other,” CoI: “other”) induced large changes in Pearson chi-square values. Covariate pattern 97 also created significant changes in deviance values. In terms of effect of a covariate pattern on coefficients of other patterns, pattern 97 again has the largest value, followed by pattern 100 (i.e., SoI: “parts and material,” PT: “alteration or rehabilitation,” CoI: “installing plumbing and lighting fixtures”). Figure 3d combines Figure 3a with Figure 3c as the size of circles represent the value of Δβ. Based on these results, three covariate patterns (i.e., 97, 55, and 100) were selected for further investigation (Table 7).

To further validate this model, one can delete each of the three questionable patterns and look at the model statistics to see if the modified model can still perform well. Columns three, four, and five of Table 7 demonstrate deviance, sum of Pearson chi-square residuals, and the Hosmer-Lemeshow goodness-of-fit statistic for three models excluding covariates 97, 55, and 100, respectively. These numbers indicate that the selected additive model (i.e., SoI + PT + CoI) can still perform well and fit the data in each case. The results of three other scenarios in Table 7 (i.e., removing two covariates with the poorest fit, removing two covariates with the largest influence, and removing all three covariates) also show that the additive model with three variables can fit the data very well in each scenario. These findings indicate that the additive model performs well with the remaining 105 covariates patterns (representing 91% of all accidents). Based on these results, the three questionable covariates were removed from the rest of analysis.

III. Validation

As mentioned by Iezzoni [63], calibration and discrimination are two main methods to assess the performance of a logistic regression model on unseen data. While discrimination measures the ability of a model to distinguish between two classes of the dependent variable, calibration determines the model’s capability in producing estimations that are, on average, close to the observed classes [64]. This study is focused on fatality rates among common accident scenarios. Due to the categorical nature of predictors, the fatality rate (i.e., dependent variable) of an accident scenario is calculated as the average of fatalities among all accidents in that scenario. For instance, among the eight accidents that have occurred in alteration/rehabilitation projects with tools and instruments representing the source of injury and interior plumbing/ducting/electrical work being the cause of injury, only one resulted in a fatality. Therefore, the observed/actual rate of fatality in this scenario is around 13%. As the objective is to study these rates and compare the performance of the logistic model to them, the authors have concluded that calibration measures would better serve this purpose.

The same measure (i.e., HL statistic) can be used for calibration/validation purposes. The only difference is that the data would be divided into training and testing sets and the model would be developed on the training set and be tested on the unseen testing set. Large p-values indicate there is no evidence to believe the model does not fit test data. For more information on the test statistics for testing dataset look at [54] (p. 155).

To validate the model on unseen data, a series of training sets were created using 70% of the source data, and a series of testing sets were created using 30% of the source data. To increase the reliability of the results, this study applied a stratified sampling method to generate training/testing data sets. The same measure (i.e., Hosmer-Lemeshow statistic) was calculated to ensure that the model, which was trained only on the training set, can fit testing data well. The chi-square statistic of 6.97 and the *p*-value of 0.540 showed that the proposed model can fit unseen data as well.

## 4. Results

This section starts by presenting the odds ratios of fatality among three significant accident factors and continues by presenting the final model, its coefficients, and the 36 most common accident patterns among electrical contractors that can be explained by the model.

### 4.1. Odds Ratio

Before presenting the final model, one can examine unadjusted odds ratios for the three variables that are included in the model: project type, source of injury, and cause of injury. The significant ratios reveal which categories of a variable caused higher fatality rates. These ratios can also be compared to adjusted ratios (i.e., controlled for other variables) from the logistic model and to identify the effect of controlling factors on each variable. Table 8 demonstrates these ratios among project types. In the first row, 1.24 means that fatal accidents in “alteration or rehabilitation” projects are 1.24 times more likely to happen than in “maintenance or repair” projects. This ratio is the same as saying that fatal accidents are 0.81 times (second row) as likely to happen in “maintenance or repair” projects than in “alteration or rehabilitation” ones. As Table 8 shows, the odds of fatal accidents in the two largest project types (i.e., “new” and “alteration”) are almost identical. Table 8 also shows that while the odds of an occupational death are much larger in “demolition” projects than “new,” “maintenance,” and “alteration” projects, the difference is not significant due to the low frequency of “demolition” projects, which, in turn, produces large confidence intervals. While the definition of the ‘Other’ category is ambiguous, since no further details were available from accident reports, it was not possible to translate this category among project types.

Table 9 demonstrates the odds ratios among “sources of injuries”. As one can see, when the source was a “tool, instrument, or equipment,” much fewer fatalities happened as compared to tasks that involved “machinery,” “parts and material,” or “vehicles.” On the contrary, “vehicles” could lead to more severe injuries than “parts and materials,” “structures and surfaces,” or “tools, instruments, and equipment.” In fact, having a fatal accident is 2, 3, and 4.4 times more likely to occur when the source is a “vehicle” than when it is a “part and material,” a part of “structure or surface,” or a “tool, instrument, or equipment,” respectively. Among sources of injuries, 56 cases were defined as “other” in OSHA’s database. After reviewing accident reports, all 56 cases were reclassified with meaningful values (vehicles, machinery, etc.). However, there were seven categories (e.g., chemical products, natural gas, containers, etc.) with very low frequencies (less than 2% of sources). To reduce the number of groups in the analysis, the authors combined these categories (accounting for 23 cases all together) into one “other” category. One should note that this “other” category is less ambiguous after content analysis as its components are known.

The odds ratios for different causes of injuries are shown in Table 10. Four ratios were found to be significant. When the tasks are “fencing, installing lights, signs, etc.” or “installing plumbing, lighting fixtures,” the likelihood of a fatal accident is 2.88, and 2.01 times the likelihood of a non-fatal accident than when the task is “interior plumbing, ducting, or electrical work” respectively. In other words, “interior plumbing, ducting, or electrical work” is less hazardous than the other two tasks. Also, “fencing, installing lights, signs” and “installing plumbing and lighting fixtures” cause fatality, respectively, 2.39 and 1.67 times more than “Other” causes. One should note that the ‘Other’ category here is consisted of two types of cases: (1) causes with low frequency incidents and (2) reports in which the cause was not reported by OSHA inspectors. The first group represented less than 5% of the data. From 34 reported causes, only five had at least 5% and the rest (e.g., demolition, excavation, cutting concrete pavement) were labeled as ‘Other’. Other than low frequency causes, there were 130 cases in which the cause of injury was not reported by OSHA inspectors. Interestingly, the fatality rate of ‘not reported’ cases was the same as those of the ‘Other’ category in the data: 32% of injuries in both groups were resulted in a fatality. For this reason, the ‘not reported’ cases were also added to the ‘Other’ category when calculating the odds ratios and building the regression model.

### 4.2. Final Logistic Regression Model

After selecting the final multi-variate model, one can discuss its coefficients for different levels of variables. Note that reference groups among variables are set to those with the highest fatality rates: accidents that have occurred in “demolition” projects, those where source of injury was a “vehicle,” and those wherein the cause was “fencing, installing lights, signs, etc.” From the 14 coefficients, six (excluding the constant) are significant at the level of 0.05, and three are marginally significant at the 0.10 significance level.

New adjusted odds ratios can be calculated easily from Table 11. For instance, to compute the adjusted odds ratios between “machinery” and “vehicle,” one can first get the estimated logits under the model:

(1) $$\hat{g}\left(SoI=Machinery;PT^{*}=Alteration\;or\;rehabilitation;CoI^{*}=Installing\;plumbing,\;lighting\;fixtures\right)=1.862-0.733-0.607-0.420=0.102;$$

(2) $$\hat{g}\left(SoI=Vehicles;PT=Alteration\;or\;rehabilitation;CoI=Installing\;plumbing,lighting\;fixtures\right)=1.862-0.607-0.420=0.835$$

* Values for PT and CoI could vary as long as same values are used in both cases

The difference between the two estimations is −0.733 and the odds ratio would be the exponential of −0.733, which is 0.48. Comparing this ratio to Table 9 reveals that controlling for “project type” and “cause of injury” has decreased the odds of fatality for machinery by around 17% (i.e., 0.58 to 0.48). Note that this odds ratio is more accurate than the unadjusted one in Table 9 (i.e., 0.58) as the effects of more accident factors are considered.

### 4.3. Common Accident Patterns among Electrical Workers

To investigate common covariate patterns and study the performance of the model in predicting the fatality rates among them, the authors decided to look into the observed and predicted fatality rates among patterns with at least six cases (i.e., representing at least one percent of the data). Using this criterion reduced the number of patterns to 36, which represent 411 accidents or 73 percent of the data. Table 12 shows these results, which were sorted based on the observed fatality rates in each pattern. Among the 36 patterns, in 11 cases, the predicted value was within 5 percent of the observed fatality rates. These 11 patterns represent 38 percent of the 411 accidents in Table 12. Another 10 patterns, representing 28 percent of the accidents, were predicted with 6–10 percent of the observed rates. Among the rest of patterns, four of them did not have close prediction numbers, but yielded ranks predicted correctly as having the highest fatality rates. For instance, the predicted fatality rate for pattern 99 was 62 percent, which is the second highest prediction rate and indicates the high risk-level of this pattern. These numbers indicate the satisfactory ability of the model to detect the level of risk among various accident patterns.

## 5. Discussion

To explore and quantify the effect of the three significant factors (i.e., “project type,” “source of injury,” and “cause of injury”) on fatality rates, odds ratios were calculated, and their significance was tested at the 0.95 level. Among “sources of injuries,” results indicate that “machinery” and “parts and materials’’ can cause death at significantly higher rates than “tools and instruments”. Moreover, in accidents where the primary “source of injury” was a “vehicle,” odds of a fatal injury would be two times, three times, and four times more than where the sources are “parts and material,” “structures and surfaces,” and “tools and instruments,” respectively. “Vehicles” in this study include highway motorized vehicles, powered off-road and industrial vehicles, and non-powered plant and industrial vehicles. All fatal accidents involving vehicles in this study can be categorized into four scenarios: (1) struck by/run over by passenger vehicles or construction vehicles; (2) electrocuted by touching part of a vehicle (e.g., boom) that was energized mistakenly; (3) fell from truck/forklift/bucket; and (4) pinned between vehicles or part of vehicles. Chi and Lin [65] have mentioned the imperative of using vehicles that are in compliance with standards such as American National Standard for Powered Industrial Trucks. They also emphasized the need for required training and evaluation of operators based on OSHA standards. For instance, to avoid falls, OSHA 1910.178 declares that operators must avoid unsafe behaviors such as placing arms or legs between the uprights of the mast or outside the running lines of the truck or standing or passing under the elevated portion of any truck. Applying these recommendations could reduce the risk of accident scenarios involving vehicles.

While the odds ratios could show which level(s) of factors are more hazardous (i.e., produce higher fatality rates), they are not enough to explain the relationship between fatality rates and accident scenarios in which more than one factor is present. In other words, considering combinations of attributing factors in a regression context could possibly reveal more about the outcome of accidents (i.e., the probability of a fatal injury in this case) than when only one factor has been studied. As mentioned by Hosmer et al. [54], the dependent variable is usually associated with more than one predictor and therefore considering only univariate models could be insufficient and misleading. Therefore, the authors took several steps to find the best fitting, most parsimonious, and efficiently interpretable multivariate logistic regression model. The final model is an additive model consisting of three factors: source of injury; type of project; and cause of injury. The analysis shows that this model is better than any of the other 17 possible models and the saturated model (Table 2 and Table 3), that it can fit the data well (Table 5 and Figure 2), and that it has a satisfactory performance on unseen data validated by Hosmer-Lemeshow test. To ensure that the model can fit individual patterns, the authors have also performed diagnostic tests, which revealed that three patterns do not fit the model. These patterns have been removed from the data.

Using coefficients of the final model (Table 11), one can study new adjusted odds ratios among factors. These ratios are different from those in Table 2, Table 3 and Table 4, as the new ones are controlled for the use of other factors in the model. For instance, controlling for “project type” and “cause of injury,” the odds ratios of “vehicles” to “machinery,” “structure and surfaces,” “tools, instruments, and equipment,” and “other” sources would increase by 20 percent, 13 percent, 13 percent, and 13 percent, respectively, while the odds ratio between “vehicles” and “parts and materials” would be reduced by 1 percent (compared to the ratios that are not adjusted for “project type” and “cause of injury” in Table 9: “vehicles’” row). The significance of coefficients strongly confirms the effects of the selected factors on the rate of fatality.

The logistic regression model can also be used to estimate the probability of a fatal injury in a specific accident scenario. Using statistical methods to find common accident scenarios have been tested in previous studies. For instance, Chi et al. [66] have applied Phi coefficients on fatal accidents to find strong positive associations between industry, sources of injury, and accident type. They reported that fatal falls from structures and construction facilities had the most obvious link to the construction industry. In this paper, the authors have considered both fatal and non-fatal accidents and used the fatality rates as the measure of risk among accident patterns.

From the 105 accident scenarios/patterns used to validate the model, 36 of them happened more frequently. Each of these scenarios occurred at least six times, and together they cover 73 percent of all accidents in this study. To identify and study more hazardous scenarios both in terms of severity and frequency, observed rates of fatality and total number of accidents in each scenario were calculated. Furthermore, the logistic model’s estimated rates of fatality were computed and compared to observed rates to detect which scenarios could be predicted correctly. The two most common patterns share the same source (i.e., “parts and materials”) and cause of injury (i.e., “other”), and only differ in their “project type.” Among the two, most accidents (32 cases) occurred in “new projects or new additions,” followed closely by “maintenance and repair” (31 cases). Considering the fatality rates, though, “new projects” have caused fewer fatalities (31 percent) than “maintenance” projects (36 percent). Without considering the source and cause of injury, the total fatality rates in “new projects” and “maintenance projects” were 40 percent and 35 percent, respectively. The reduction in fatality rates among “new projects” (when putting source and cause into picture) is notable. One can conclude that when the “source of injury” is a “part or material” (e.g., an electrical part) and the cause is “other,” “new projects” are much less hazardous than “maintenance projects.”

In terms of severity, there were nine patterns with at least 50 percent fatality rates. Among them, in five patterns the “source of injury” was “parts and materials”: two “alteration projects,” two “new projects,” and one “maintenance.” Reviewing the fatality rates among these five patterns further confirms the effect of “cause of injury” on fatality rates. Note that with the same source (i.e., “parts and material”) a different cause (i.e., “fencing, installing lights, signs, etc.”) increased the fatality rates from 36 percent to 67 percent in “maintenance projects” (Table 12). “Type of project” can also play a significant role in determining the fatality rates. For instance, when “source of injury” was a “part or material” and “cause” is “installing equipment,” the fatality rates could vary from 17 percent in other projects to 59 percent in “alteration or rehabilitation projects.” These jumps in probabilities signal that safety managers may benefit from preemptively addressing such coinciding factors in work situations to plan around the risks and thereby offset them.

Considering the prediction performance, while the range of actual fatality rates among 36 patterns was 0 to 83, the predicted rates represent a smaller range of 5 to 62 percent. This difference is important in interpreting the results as, for instance, an estimated value of 62 percent would refer to the most dangerous patterns whereas the same value might not be as alarming among observed rates. One should note that while this smaller range caused some large variations between the observed and estimated values—especially among larger rates (i.e., greater than 50 percent)—the level of severity was predicted correctly in these cases. As an example, consider the most fatal pattern (83 percent death rate), which occurred in “alteration projects” when the “source” and “cause of injuries” were “part and materials” and “fencing, installing lights, signs, etc.,” respectively. The predicted rate for this pattern (i.e., 62 percent) was the second highest predicted rate, which clearly declares the high risk of this accident pattern. The research team could identify four of such patterns in which the predicted rate—thought not close to actual rates—accurately determined the high-risk level of the pattern. Other than that, 11 and 10 scenarios were estimated within 5 percent and 10 percent of the actual rates, respectively. These 25 patterns represent 69 percent of the patterns and 73 percent of the accidents.

Another practical application of the results is related to risk assessment of projects for electrical contractors. For example, as shown in Table 12, in new projects or new addition projects, when workers were “installing plumbing, lighting fixtures” and exposed to “parts and materials”, the risk of fatality was the highest. In another example scenario, if the project was “alteration or rehabilitation” and activity was “fencing, installing lights, signs, etc.”, the risk of fatality was the highest again when workers were exposed to “parts and materials”. In these cases, a project or safety manager can assign more safety resources, conducting job hazard analysis, or toolbox meetings to increase awareness of construction workers regarding potential hazards.

## 6. Conclusions

Electrical contractors working in the construction industry are exposed to various hazardous situations leading to a high number of severe injuries and fatalities [67]. This problem necessitates the identification of high-priority accident scenarios both in terms of frequency and severity (i.e., probability of a fatal outcome) to thereby allow for risk mitigation. Accordingly, the objective of the present work was to first study individual effects of different contributing factors (e.g., project characteristics, sources and causes of accident) on degree of an injury and then explain the significant effects through a multivariate logistic model. Rather than estimating the probability of a single accident, the authors utilized three significant factors (i.e., “project type,” “source of injury,” and “cause of injury”) to form 108 accident scenarios (i.e., covariate patterns) and reviewed these scenarios both in terms of frequency and fatality rates. Model assessment techniques verified the fit of the model to the data and its capability to estimate the probability of a fatal injury in future cases. All analysis has been done using R language packages [15].

Implications of this study suggest that, when controlling for “project type” and “cause of injury,” “vehicles” cause significantly higher fatality rates than “tools, instruments, and equipment,” “structures and surfaces,” “parts and materials,” and to a lower degree “machinery.” Considering common accident scenarios (i.e., 36 scenarios that were repeated at least 6 times in the data), there were nine of patterns with fatality rates of 50 percent or more. The logistic model also assigned the highest probabilities to most of these scenarios, demonstrating the effectiveness of our approach to predictive accident modeling. These patterns should be considered as high-priority safety-risk scenarios by electrical contractors and their safety managers when allocating safety resources and planning for possible interventions. Outside of these practical implications, this study also contributes to the body of knowledge by providing a roadmap for building multivariate regression models for safety studies.

This work has discussed several contributing factors to analyze accidents that occur to electrical contractors. Future studies can incorporate more variables such as “time of the accident” to develop models with better prediction performance. The severity of accidents also can be defined more accurately by considering more variables such as “monetary cost of injuries” or “days away from work” for non-fatal incidents. Furthermore, more advanced, non-parametric machine learning methods can be applied to similar data for the same classification purposes. Addressing these limitations can improve the prediction ability of models in accident-severity classification problems.

## Figures and Tables

**Table 1:** Fitting univariate logistic models.

Model	G	Degree of Freedom (d.f.)	*p*-Value
End use (EU)	0.4	4	0.983
Project type (PT)	18.7	4	0.001 *
Project cost (PC)	6.36	6	0.384
Source of injury (SoI)	21.58	5	0.001 *
Cause of injury (CoI)	11.80	5	0.038 *

* Statistically significant variables at 0.25.

**Table 2:** Deviance for logit models of fatality by “source of injury,” “project group,” and “project cost”.

ID	Model	Logit (π_ijk_)	Deviance	d.f.
1	Null	*η*	156.87	107
	One Factor			
2	SoI	*η + α_i_*	135.29	102
3	PT	*η + β_j_*	138.16	103
4	CoI	*η* + *γ_k_*	145.07	102
	Two Factors			
5	SoI + PT	*η* + *α_i_* + *β_j_*	113.59	98
6	SoI + CoI	*η* + *α_i_* + *γ_k_*	123.40	97
7	PT + CoI	*η* + *β_j_* + *γ_k_*	125.49	98
8	SoI × PT	*η* + *α_i_* + *β_j_* + *(αβ)_ij_*	91.65	80
9	SoI × CoI	*η* + *α_i_* + *γ_k_* + *(αγ)_ik_*	98.64	72
10	PT × CoI	*η* + *β_j_* + *γ_k_* + *(βγ)_jk_*	100.98	82
	Three Factors			
11	SoI + PT + CoI	*η* + *α_i_* + *β_j_* + *γ_k_*	101.20	93
12	SoI × PT + CoI	*η* + *α_i_* + *β_j_* + *γ_k_* + *(αβ)_ij_*	78.21	75
13	SoI × CoI + PT	*η* + *α_i_* + *β_j_* + *γ_k_* + *(αγ)_ik_*	75.10	68
14	SoI + PT × CoI	*η* + *α_i_* + *β_j_* + *γ_k_* + *(βγ)_jk_*	77.79	77
15	SoI × PT + SoI×CoI	*η* + *α_i_* + *β_j_* + *γ_k_* + *(αβ)_ij_* + *(αγ)_ik_*	55.17	50
16	SoI × PT + PT × CoI	*η* + *α_i_* + *β_j_* + *γ_k_* + *(αβ)_ij_* + *(βγ)_jk_*	54.38	59
17	SoI × CoI + PT × CoI	*η* + *α_i_* + *β_j_* + *γ_k_* + *(αγ)_ik_* + *(βγ)_jk_*	56.75	52
18	SoI × PT + SoI × CoI + PT × CoI	*η* + *α_i_* + *β_j_* + *γ_k_* + *(αβ)_ij_* + *(αγ)_ik_* + *(βγ)_jk_*	37.55	34

**Table 3:** Model comparisons.

Test ID	Test (Model A vs. Model B)	G	d.f.	*p*-Value
a1	11 vs. 5	12.39	5	0.030
a2	11 vs. 6	22.20	4	0.000
a3	11 vs. 7	24.29	5	0.000
b1	12 vs. 11	22.99	18	0.191
b2	13 vs. 11	26.1	25	0.402
b3	14 vs. 11	23.41	16	0.103
c1	15 vs. 11	46.03	43	0.348
c2	16 vs. 11	46.82	34	0.071
c3	17 vs. 11	44.45	41	0.329
d1	18 vs. 11	63.65	59	0.316

**Table 4:** Additive models with and without non-significant variables.

Model	G	d.f.	*p*-Value
PT + SoI + CoI + EU	0.73	4	0.949
PT + SoI + CoI + PC	4.73	6	0.579
PT + SoI + CoI + EU + PC	5.51	10	0.855

**Table 5:** Observed and estimated frequencies within ten deciles of risk for fatal and non-fatal accidents.

Decile	Cut Point	Non-Fatal		Fatal	
		Observed	Expected	Observed	Expected
1	[0.058, 0.186]	55	56.4	10	8.6
2	(0.186, 0.255]	54	51.3	12	14.8
3	(0.255, 0.295]	48	50.8	23	20.2
4	(0.295, 0.310]	32	32.0	14	14.0
5	(0.310, 0.364]	48	52.3	32	27.7
6	(0.364, 0.390]	35	31.1	15	18.9
7	(0.390, 0.402]	38	33.7	18	22.3
8	(0.402, 0.499]	33	33.0	29	29.0
9	(0.499, 0.561]	26	29.1	35	31.9
10	(0.561, 0.781]	24	23.3	38	38.8

**Table 6:** Covariate values, observed outcomes (y_ijk_), number of cases in the covariate pattern (m_ijk_), observed probability (P_ijk_), estimated logistic probability (π_ijk_), and the value of four diagnostic statistics Δχ^2^ (i.e., change in value of the Pearson chi-square), ΔD (i.e., change in the deviance), h (i.e., leverage), and Δβ (i.e., change in value of the estimated coefficients), for three covariate patterns with at least one large diagnostic value.

Covariate Pattern Number	97	55	100
Source of injury	Parts and materials	Machinery	Parts and materials
Project type	Alteration or rehabilitation	Other	Alteration or rehabilitation
Cause of injury	Other	Other	Installing plumbing, lighting fixtures
y_jjk_	5	2	14
m_jjk_	28	4	21
P_ijk_	0.179	0.500	0.667
π_ijk_	0.373	0.149	0.524
Δχ^2^	6.667	4.464	2.737
ΔD	7.452	3.115	2.802
h	0.322	0.125	0.377
Δβ	3.166	0.639	1.658

**Table 7:** Model statistics for the original model and six scenarios.

Model Statistics	All Data Coefficients	97	55	100	Poorest Fit (97, 55)	Largest Influence (97, 100)	All Three
D	101.20	94.02	97.90	98.42	90.81	92.68	89.67
Χ^2^	83.82	77.24	77.24	80.99	74.35	75.62	72.94
C	3.17	1.60	1.01	4.21	3.03	2.92	2.24

**Table 8:** Odds ratios of fatal accidents among different project types.

Project Types	Alteration or Rehabilitation	Maintenance or Repair	New Project or New Addition	Demolition	Other
Alteration or rehabilitation ^†^	-	1.24	1.01	0.47	4.06 *
Maintenance or repair ^†^	0.81	-	0.81	0.38	3.28 *
New project or new addition ^†^	0.99	1.23	-	0.47	4.02 *
Demolition ^†^	2.11	2.62	2.13	-	8.58 *
Other ^†^	0.25 *	0.25 *	0.42 *	0.12 *	-

* Statistically significant at 0.05 level; ^†^ First term in odds ratio.

**Table 9:** Odds ratios of fatal accidents among different sources of injuries.

Source of Injury	Machinery	Parts and Material	Structures and Surfaces	Tools and Instruments	Vehicles	Other
Machinery ^†^	-	1.26	1.79	2.57 *	0.58	1.50
Parts and Material ^†^	0.79	-	1.42	2.04 *	0.46 *	1.19
Structures and Surfaces ^†^	0.56	0.71	-	1.44	0.32 *	0.84
Tools and instruments ^†^	0.39 *	0.49 *	0.70	-	0.23 *	0.58
Vehicles ^†^	1.73	2.18 *	3.08 *	4.44 *	-	2.59
Other ^†^	0.67	0.84	1.19	1.71	0.39	-

* Statistically significant at 0.05 level, ^†^ First term in odds ratio.

**Table 10:** Odds ratios of fatal accidents among different causes of injuries.

Cause of Injury	Fencing, Installing Lights, Signs	Installing Equipment	Installing Plumbing	Interior Plumbing, Ducting	Temporary Work	Other
Fencing, installing lights, signs ^†^	-	1.74	1.43	2.88 *	1.52	2.39 *
Installing equipment ^†^	0.58	-	0.82	1.66	0.88	1.37
Installing plumbing ^†^	0.70	1.22	-	2.01 *	1.07	1.67 *
Interior plumbing, ducting ^†^	0.35 *	0.60	0.50 *	-	0.53	0.83
Temporary work ^†^	0.66	1.41	0.94	1.89	-	1.57
Other ^†^	0.42 *	0.73	0.60 *	1.21	0.64	-

* Statistically significant at 0.05 level, ^†^ First term in odds ratio.

**Table 11:** Final model for the electrical contractors (number of remaining accidents = 566).

Accident Factor	Model Parameters	Coefficient	Std. Err.	z	*p*
	Constant	1.862	0.786	2.369	0.018 *
SoI	Machinery	−0.733	0.445	−1.646	0.100 ^†^
	Parts and materials	−0.764	0.336	−2.276	0.023 *
	Structures and surfaces	−1.247	0.381	−3.272	0.001 **
	Tools, instruments, and equipment	−1.613	0.381	−4.235	2.29 × 10^−5^ ***
	Other	−1.072	0.545	−1.966	0.049 *
PT	Alteration or rehabilitation	−0.607	0.651	−0.933	0.351
	New project or new addition	−0.635	0.629	−1.009	0.313
	Maintenance or repair	−1.082	0.637	−1.699	0.089 ^†^
	Other	−2.456	0.751	−3.268	0.001 **
CoI	Installing equipment (HVAC and other)	0.445	0.435	−1.023	0.306
	Installing plumbing, lighting fixtures	−0.420	0.476	−0.883	0.377
	Interior plumbing, ducting, electrical work	−1.032	0.450	−2.290	0.022 *
	Temporary work (buildings, facilities)	−0.389	0.526	−0.740	0.459
	Other	−0.735	0.421	−1.748	0.081 ^†^

Significance levels: 0–0.001: ***; 0.001–0.01: **; 0.01–0.05: *; 0.05–0.1: ^†^. HVAC: heating, ventilation, and air conditioning.

**Table 12:** Observed and predicted probability of fatal accidents in 36 common covariate patterns.

ID	Observed	Estimate	SoI	Cause	Type	*n*
149	0.00	0.07	Structures and surfaces	Other	Other	6
137	0.00	0.23	Structures and surfaces	Other	Maintenance or repair	6
165	0.10	0.22	Tools, instruments, and equipment	Installing plumbing, lighting fixtures	Maintenance or repair	10
160	0.13	0.20	Tools, instruments, and equipment	Interior plumbing, ducting, electrical work	Alteration or rehabilitation	8
106	0.15	0.27	Parts and materials	Interior plumbing, ducting, electrical work	Maintenance or repair	13
179	0.17	0.05	Tools, instruments, and equipment	Other	Other	6
116	0.17	0.14	Parts and materials	Installing equipment (HVAC and other)	Other	6
173	0.17	0.25	Tools, instruments, and equipment	Other	New project or new addition	12
119	0.20	0.11	Parts and materials	Other	Other	10
172	0.27	0.20	Tools, instruments, and equipment	Interior plumbing, ducting, electrical work	New project or new addition	11
158	0.29	0.31	Tools, instruments, and equipment	Installing equipment (HVAC and other)	Alteration or rehabilitation	7
170	0.31	0.30	Tools, instruments, and equipment	Installing equipment (HVAC and other)	New project or new addition	13
113	0.31	0.43	Parts and materials	Other	New project or new addition	32
112	0.33	0.36	Parts and materials	Interior plumbing, ducting, electrical work	New project or new addition	24
108	0.33	0.41	Parts and materials	Temporary work (buildings, facilities)	Maintenance or repair	9
11	0.33	0.63	Vehicle	Other	Alteration or rehabilitation	6
143	0.35	0.32	Structures and surfaces	Other	New project or new addition	26
107	0.36	0.33	Parts and materials	Other	Maintenance or repair	31
171	0.36	0.31	Tools, instruments, and equipment	Installing plumbing, lighting fixtures	New project or new addition	11
104	0.36	0.39	Parts and materials	Installing equipment (HVAC and other)	Maintenance or repair	11
167	0.38	0.17	Tools, instruments, and equipment	Other	Maintenance or repair	8
161	0.38	0.25	Tools, instruments, and equipment	Other	Alteration or rehabilitation	8
142	0.38	0.26	Structures and surfaces	Interior plumbing, ducting, electrical work	New project or new addition	8
159	0.38	0.32	Tools, instruments, and equipment	Installing plumbing, lighting fixtures	Alteration or rehabilitation	8
110	0.41	0.51	Parts and materials	Installing equipment (HVAC and other)	New project or new addition	17
140	0.42	0.39	Structures and surfaces	Installing equipment (HVAC and other)	New project or new addition	12
101	0.47	0.37	Parts and materials	Interior plumbing, ducting, electrical work	Alteration or rehabilitation	17
47	0.50	0.33	Machinery	Other	Maintenance or repair	6
17	0.50	0.51	Vehicle	Other	Maintenance or repair	6
128	0.57	0.39	Structures and surfaces	Installing equipment (HVAC and other)	Alteration or rehabilitation	7
98	0.59	0.51	Parts and materials	Installing equipment (HVAC and other)	Alteration or rehabilitation	17
103	0.67	0.50	Parts and materials	Fencing, installing lights, signs, etc.	Maintenance or repair	9
114	0.67	0.52	Parts and materials	Temporary work (buildings, facilities)	New project or new addition	6
23	0.67	0.62	Vehicle	Other	New project or new addition	9
111	0.78	0.51	Parts and materials	Installing plumbing, lighting fixtures	New project or new addition	9
99	0.83	0.62	Parts and materials	Fencing, installing lights, signs, etc.	Alteration or rehabilitation	6
